# Annual Meeting of the State Medical Society

**Published:** 1881-04

**Authors:** 


THE ANNUAL MEETING OF THE STATE MEDICAL SOCIETY (Medical and Chirurgical) was held in this city from the 12th to the 15th instant inclusive. Interesting lectures were delivered by Prof. Martin, of the Johns Hopkins University, and others on the 13th. On the 14th Prof. William Goodell, of the University of Pennsylvania, delivered the annual oration, and our Health Commissioner read an elaborate paper on hygiene. The Society honored itself by electing Prof. Frank Donaldson, M. D., to its Presidency for the ensuing year.

